# Attitudes of the police towards individuals with a known psychiatric diagnosis

**DOI:** 10.1186/s12888-022-04234-1

**Published:** 2022-09-19

**Authors:** M. Mengual-Pujante, I. Morán-Sánchez, A. Luna-Ruiz Cabello, M. D. Pérez-Cárceles

**Affiliations:** 1 Department of Legal and Forensic Medicine, Regional Campus of International Excellence “Campus Mare Nostrum”, Faculty of Medicine, University of Murcia, Murcia, Spain; 2 Cartagena Mental Health Centre, Real St 8, E-30201, Cartagena, Murcia, Spain; 3 Department of Forensic Psychiatry, Law Faculty, Catholic University of Murcia, Murcia, Spain

**Keywords:** Stigma, Stereotypes, Police, Schizophrenia, Depression, Forensic psychiatry

## Abstract

**Background:**

Police officers are increasingly required to respond to incidents involving psychiatric patients. However, few studies have assessed whether the attitude of police officers depends on prior knowledge of their specific psychiatric diagnosis. Our aim was to analyze the effects of psychiatric diagnosis on the behavior of police officers.

**Methods:**

We utilized the Attribution Questionnaire adapted to the police context to examine the attitudes of 927 officers of the Spanish National Police Force towards persons diagnosed with either schizophrenia or depressive disorder playing the role of somebody in need of assistance, a victim of a crime, a witness, or a suspect in a criminal case. Different socio-demographic variables were also collected.

**Results:**

Compared to attitudes to individuals with a known psychiatric diagnosis, police officers expressed increased willingness to help psychiatric patients and increased sympathy and attributing to them less responsibility for their actions. They also showed increased feelings of avoidance, reported a greater perception of danger and a greater need for isolation and involuntary treatment. This was especially so in the case of schizophrenia. Stigmatizing attitudes were less apparent when the person was a woman, a veteran officer, or someone with a history of work experience.

**Conclusions:**

Police officers may hold certain stigmatizing attitudes towards persons with mental illness, particularly schizophrenia, that require special attention, as they may negatively affect police action. We found several factors associated with the persistence of these stigmatizing attitudes among police officers that may guide us when implementing training programs for promoting attitude change, especially at the beginning of an officer’s professional career.

## Background

Over the last 20 years, in Spain and in other European countries, there has been a significant development and improvement in mental health services as a result of various health care policy changes. There has been a transition from institutional care of people with mental illnesses to family and community-based care. Most regions in Spain have their own mental health plans that support social inclusion, independent living, employment, and human rights [1]. Despite the progress made in this area, society in general, and even health professionals themselves, still maintain certain negative attitudes that may lead to the rejection and social isolation of psychiatric patients [2–8]. Recent studies suggest that professional encounters between the police and persons with mental illness have increased considerably in recent years [9–11]. For instance, in the United Kingdom, approximately 2% of incidents reported to the police over a one-year period were linked to mental health issues [11]. Post deinstitutionalization, police officers are increasingly required to respond to incidents in the community involving mentally ill people [10, 12, 13]. Police officers may identify mental health calls and are often the first responders in mental health crises [9, 11, 13, 14]. They play an important role in helping to facilitate access to appropriate health care and treatment, by partnering with other emergency medical providers [15]. For example, in Spain, transfer protocols have been set up involving the healthcare services and police officers [16]. These include training in advanced communication skills that assist in the de-escalation of mental health incidents [13].

This leads us to study the attitudes of the police during mental health-related encounters since, if these are negative, they could present an obstacle to the exercise of the defense of the rights and protection of psychiatric patients. It is for these reasons that, over the last few years, there has been a growing trend towards assessing stigma among police officers when they encounter psychiatric patients [17]. Previous studies into this phenomenon mostly reflect that police officers tend to hold the same points of view about psychiatric patients as the general population, only somewhat more negative [13, 17, 18]. For example, Watson et al. found that police officers often questioned the credibility of patients with schizophrenia and considered them to be more dangerous than people without a mental illness diagnosis. The label of schizophrenia was also associated with a greater desire to help, greater sympathy and a lesser inclination to hold patients with schizophrenia responsible for their acts [17]. Another study found that police officers, more than the general public and more than psychiatrists, perceived psychiatric patients to be unpredictable, dangerous, and difficult to manage. They also feared them more than members of the public or psychiatrists [18]. The limitations of these studies include small the sample sizes, the scarce sociodemographic background information, the heterogeneity of assessment instruments and the undifferentiated role of the psychiatric patient during interactions with police officers. On the other hand, most of these studies assessed attitudes towards only one type of mental illness, usually schizophrenia, while failing to explore other common and prevalent disorders such as depression. Furthermore, most of the research was carried out in the USA and Canada [18–20], and, to a lesser extent, in Europe [21, 22], which makes it difficult to extrapolate the results to the Spanish police context. To the best of our knowledge, no previous study has evaluated police attitudes towards mental illness in the Spanish context. Therefore, we set out to assess how awareness of a person’s mental illness influences members of the Spanish police forces. Our hypothesis was that mental health stigma would be evident regardless of the role of the psychiatric patient during the interaction with police officers. We hypothesized that police officers would perceive psychiatric patients to be less credible and more dangerous than someone behaving in an identical manner but who had not been identified as someone with a mental illness. We predicted that stigma would be worse for schizophrenia than for depressive disorder and it would manifest as a stronger likelihood to isolate the person and to insist on treatment and, also, to avoid the person. A further prediction was that the label of mental illness would increase a police officer’s willingness to help the person, would evoke feelings of sympathy, and would make the person seem less responsible for his or her actions.

## Methods

### Type of study

A cross-sectional study was conducted and approved by the Research Ethics Committee of the University of Murcia and was authorized by the Training and Development Division within the Directorate General of the Police.

### Participants

The study was conducted at the National Police School in Ávila, a city in the center of Spain where all the training courses of the institution take place. Members of the Spanish police forces who wish to be promoted to a higher rank must fulfill several requirements, such as passing a national exam, undergoing a 10-month training period, and having a certain minimum length of service. For example, to be promoted from sub-inspector to inspector requires a length of service of at least 5 years.

### Procedures

The research was carried out according to the instructions set out in the authorization sent by the Training and Development Division. Before starting the fieldwork, several coordination meetings between a member of the research team and the Heads of Studies of the Spanish National Police took place. Those officers who had attended different training courses during the previous year were invited to participate by a member of the research team who explained the study to them during the training courses. They carefully reviewed the study information together and an informed consent form was signed. The research staff explained that the study was about police attitudes and decisions and that it was intended for training purposes – the emphasis on mental illness was not mentioned. The officers who wished to participate were asked to complete a questionnaire over a period of one month. At the end of the deadline, all the questionnaires were collected.

### Measurements

Data were gathered from each participant by means of a questionnaire designed to ascertain the socio-demographic variables shown in previous research to influence the attitudes of a police officer toward those with mental illness: gender, age, educational level, familiarity with mental illness, and number/frequency of encounters with mentally ill people [23].

To assess how the stereotype of mental illness may influence the behavior of the participants, vignettes designed by Watson et al. were used [17]. Spanish translations of these vignettes are available upon request. These neutral vignettes describe a hypothetical subject in the role of a person in different police situations, i.e., a person in need of assistance, a victim, a witness, or a suspect. The hypothetical subject, called Pedro, is described as suffering either from schizophrenia or a depressive disorder, or to have no psychiatric diagnosis. The vignettes do not describe any behavior or physical description of the fictitious protagonist, as their aim is to determine whether and how the label of a mental illness influences the response of police officers [17]. The vignettes are designed not to portray serious infractions, allowing the police officers the maximum possible discretion in responding. Otherwise, they would have to act in accordance with the Spanish criminal code, thereby limiting their responses. Officers were randomly assigned one of twelve versions of the survey: one for each of Pedro’s four different roles (person in need of assistance, victim, witness, or suspect), plus label of schizophrenia, depressive disorder or no mental illness. We chose those diagnoses because they are the ones most frequently encountered by police officers.

Stigmatizing attitudes and beliefs about mental illness were assessed using the Attribution Questionnaire-27 (AQ-27) designed by Corrigan et al. [24] and modified by Watson et al. [25]. This tool has been translated into many languages [26–28]. The study used the validated Spanish version of the modified scale developed by Muñoz et al. [28]. This self-administered questionnaire consists of 31 items grouped into nine factors/dimensions, each assessing the following stigma-related constructs: *responsibility*, *pity*, *anger*, *dangerousness*, *help*, *coercion*, *segregation*, *credibility*, and *avoidance*. Participants were asked to rate their level of agreement with each statement on a Likert scale ranging from 1 (“not at all”) to 5 (“very much”). Each dimension score shows the mean score of the respective items (the items of avoidance are reversed in the Spanish version). Higher scores indicate greater stigmatization.

### Statistical analysis

The statistical analysis was conducted using the software program Stata 17®. *P*-values < 0.05 were considered to be statistically significant.

First, we tested normality by P-P plots and homogeneity of variances with Levene’s test in order to see if parametric tests could be applied. As the assumptions were met, analysis of variance (ANOVA)/ t test were used.

Second, we described officers´ socio-demographic and working characteristics. We reported categorical variables (gender, civil status, education level, professional status, age range, encounter frequency and familiarity with mental illness) as frequencies and valid percentages and continuous variables (age, length of duty, academy and previous year training and number of weekly encounters with mentally ill people) as mean and standard deviations (SD) for the whole sample. The mean scores and SD of the scale measuring stigmatizing attitudes and beliefs about mental illness (the AQ scale) were calculated according to sociodemographic characteristics. Any sociodemographic related differences were tested by applying t test in binary variables, (for example, gender) or by a one-way ANOVA with post hoc Bonferroni comparisons, in variables with more than two categories (age range). Correlations between quantitative variables (age, length of duty, academy and previous year training, number of weekly encounters with mentally ill people) and AQ scores were also examined using the Pearson coefficient.

Third, we analyzed differences in stigmatizing attitudes according to mental illness. Two one-way multivariate analysis of variance **(**MANOVA**)** were conducted to determine if there were any differences between two or more independent groups of a categorical independent variable in terms of two or more continuous dependent variables. In the first MANOVA test, the vignette role, the psychiatric history (yes/no) and the interaction between them were taken as independent variables and the nine AQ subscale scores were taken as dependent variables. In the second MANOVA test**,** the independent variables were the vignette roles and the type of mental illness. To understand which independent groups were different at the univariate level (where the differences were between each of the AQ subscales scores separately), subsequent ANOVA tests were run.

Four: to test differences in stigmatizing attitudes according to mental illness, we first looked at the differences in AQ scores (the dependent variable, a continuous measure) between no mental illness vs mental illness (independent variable, a qualitative variable) and afterwards we describe in detail differences between schizophrenia vs. depression by applying a one-way ANOVA with post hoc Bonferroni comparisons. In the first step we fused schizophrenia and depression in “mental illness”.

Five: we analyzed the association between vignette role and stigmatizing attitudes**.** To test differences in AQ scores between the different vignette roles a comparative ANOVA test for each AQ factor was run. In order to see where the differences occurred, post hoc Bonferroni comparisons (assistance vs. victim vs. witness vs. suspect), were conducted.

The sample size was calculated applying the statistical treatment for quantitative variables on finite populations: for a prevalence of 50%, an error precision of 3.5% and a confidence level of 95%, the recommended sample would be 783 subjects. To avoid possible losses, a total of 1090 surveys were handed out, 163 officers refused to participate (15%); and 927 officers finally completed the study.

## Results

The baseline characteristics of the participants are shown in Table 1. The mean age of the officers was 35.5 (SD = 6.68) years, and 82.4% were male. The mean length of service was 11.26 (SD = 7.15) years. Of the group, 72.1% (*n* = 668) had had previous contact with mentally ill persons and 51.2% (*n* = 478) had experienced such encounters in their professional lives.

**Table 1:** Baseline characteristics of participants (*n* = 927)

	Category	Number of cases	Percentage
**Gender**			
	Men	764	82.4
	Women	163	17.6
**Civil status**			
	Single	451	48.7
	Married/cohabiting	427	46
	Previously married	49	5.3
**Educational level**			
	Secondary	46.5	431
	University	53.5	496
**Professional status**			
	Officer	395	42.6
	Sub-Inspector	240	25.9
	Inspector	235	25
	Chief Inspector	37	4
	Commissioner	20	2.2
	1st year executive student	41	4.4
	2nd year executive student	22	2.4
**Age range**			
	Under 31	218	23.5
	31–40	526	56.7
	Over 41	183	19.7
**Familiarity with mental illness**			
	Yes	668	72.1
	No	259	27.9
**Encounter frequency**			
	Rarely	452	48.8
	Sometimes	309	33.3
	Often	169	17.9
		**Mean (SD)**	
**Age**		35.5 (6.68)	
**Length of duty**		11.26 (7.15)	
**Academy training**		10.95 (26.02)	
**Previous year training**		13.11 (18.7)	
**Weekly encounters with mentally ill people**		3.15 (4.00)	

*SD* standard deviation

The 927 participants completed the modified AQ questionnaire after reading one of the vignettes. 28.2% (*n* = 262) received a vignette of a hypothetical person in need of assistance, 25.8% (*n* = 239) a vignette of a victim, 23.2% (*n* = 215) a vignette of a witness, and 22.8% (*n* = 211) a vignette of a suspect. 34.3% (*n* = 318) received a vignette of a person diagnosed with schizophrenia, 32.7% (*n* = 303) a vignette of a person diagnosed with depression and, 33.0% (*n* = 306) received a vignette with no mention of mental illness.

### Association between socio-demographics variables and AQ scores

When the hypothetical person, Pedro, had a history of psychiatric illness, we found that female police officers showed greater feelings of pity and desire to help than male officers: 3.24 (SD = 0.80) vs. 3.02 (SD = 0.72) points; (t = − 2.81, *p* = 0.005) and 3.99 (SD = 0.86) vs. 3.77 (SD = 0.84) points; (t = − 2.43, *p* = 0.016), respectively. Younger officers had a greater perception of danger than older officers: 2.56 (SD = 0.62) vs. 2.52 (SD = 0.61) vs. 2.38 (SD = 0.59) points respectively; F (2,895) = 5.06, *p* = 0.007). Post hoc paired Bonferroni comparison revealed significant differences for officers under 31 years vs. officers older than 41 years (*p* = 0.001), and for officers between 31 and 40 years and older than 41 years (*p* = 0.001). Officers under 31 years wished to avoid psychiatric patients less than officers between 31 and 41 years 3.30 (SD = 0.69) vs. 3.32 (SD = 0.69) points and more than older officers 3.08 (SD = 0.85) points, F (2,891) = 4.32, *p* = 0.001). Post hoc paired Bonferroni comparisons, revealed significant differences for officers under 31 years vs. officers older than 41 years (*p* = 0.002), and between officers between 31 and 41 years and older than 41 years, (*p* = 0.003). Staff members who were more familiar with mental health problems had more feelings of *pity* and a desire to *help* 2.99 (SD = 0.78) vs. 2.83 (SD = 0.79) points; t = 2.70, *p* = 0.007) and 3.78 (SD = 0.89) vs. 3.62 (SD = 0.88) points; t = 2.52, *p* = 0.012), respectively. Length of service was negatively correlated with desire for *avoidance* (*r* = − 0.102; *p* = 0.02) and perception of *dangerousness* (*r* = − 0.120; *p* <  0.001). We found no other statistically significant differences.

### Association between psychiatric history and AQ scores according to vignette role

The differences between the AQ dimensions according to vignette role and psychiatric history are shown in Table 2. All factors except *credibility* showed statistically significant differences for the different vignette roles.

**Table 2:** Attribution Questionnaire factor scores according to vignette role and psychiatric history

Factors		Assistance	***p***^a^	Victim	***p***^a^	Witness	***p***^a^	Suspect	***p***^a^
Responsibility Mean (SD)	No mental illness Mental illness	2.24 (0.68) 2.00 (0.61)	0.007	2.21 (0.56) 2.06 (0.51)	0.069	2.29 (0.62) 2.04 (0.61)	0.008	2.43 (0.67) 2.06 (0.55)	< 0.001
Pity Mean (SD)	No mental illness Mental illness	3.24 (0.60) 3.36 (0.75)	0.16	2.48 (0.81) 2.90 (0.68)	< 0.001	2.34 (0.77) 2.96 (0.69)	< 0.001	2.62 (0.81) 2.99 (0.76)	0.001
Anger Mean (SD)	No mental illness Mental illness	1.49 (0.66) 1.57 (0.62)	0.001	1.58 (0.65) 1.85 (0.79)	0.014	1.60 (0.63) 1.72 (0.68)	0.241	1.84 (0.93) 1.83 (0.82)	0.952
Dangerousness Mean (SD)	No mental illness Mental illness	2.33 (0.55) 2.55 (0.55)	0.003	2.07 (0.63) 2.48 (0.58)	< 0.001	2.13 (0.62) 2.34 (0.56)	0.016	2.38 (0.66) 2.66 (0.61)	0.003
Help Mean (SD)	No mental illness Mental illness	3.96 (0.85) 4.01 (0.84)	0.661	3.50 (0.88) 3.62 (0.87)	0.333	3.29 (1.04) 3.82 (0.82)	< 0.001	3.48 (0.89) 3.79 (0.83)	0.014
Coercion Mean (SD)	No mental illness Mental illness	2.56 (0.85) 3.36 (0.84)	< 0.001	2.16 (0.77) 3.01 (0.82)	< 0.001	1.88 (0.84) 2.74 (0.92)	< 0.001	2.24 (0.88) 3.29 (0.87)	< 0.001
Segregation Mean (SD)	No mental illness Mental illness	2.06 (0.69) 3.36 (0.84)	< 0.001	1.91 (0.76) 2.49 (0.78)	< 0.001	1.69 (0.70) 2.10 (0.74)	< 0.001	2.13 (0.85) 2.79 (0.76)	< 0.001
Avoidance Mean (SD)	No mental illness Mental illness	3.10 (0.73) 3.26 (0.75)	0.11	2.90 (0.87) 3.27 (0.73)	0.001	2.69 (0.75) 3.09 (0.73)	< 0.001	3.33 (0.74) 3.50 (0.75)	0.128
Credibility Mean (SD)	No mental illness Mental illness	3.20 (0.67) 3.15 (0.69)	0.513	2.92 (0.63) 2.81 (0.57)	0.244	3.35 (0.54) 3.23 (0.61)	0.178	2.74 (0.51) 2.72 (0.49)	0.760

*SD* standard deviation

^a^ Student t

A MANOVA test was conducted to examine the main and interaction effects of psychiatric history (yes/no) and vignette role on all subscales of the AQ. The results indicate significant main effects for psychiatric history *F* (7,863) = 9.40, *p* <  0.001) and vignette role F (3,863) =12.06, *p* <  0.001) but not for the interaction between them *F* (3,863) =1.41, *p* = 0.08.

To test the differences between the questionnaire constructs according to the vignette role and the type of mental illness, a MANOVA test was performed. The results indicate significant main effects for the type of mental illness *F* (2,863) =14.36, *p* <  0.001), and vignette role *F* (3,863) =12.79, *p* <  0.001), but not for the interaction between them *F* (6,852) =1.14, *p =* 0.222).

### Association between psychiatric history and AQ scores

Table 3 summarizes the relationship between the modified AQ scores and psychiatric history. All factors except for *anger* and *credibility* were significantly different. When no background information was available, the dimensions with the highest overall mean scores were *help* and *credibility,* with 3.58 (SD = 0.95) and 3.06 (SD = 0.64) points, respectively. When the hypothetical subject had schizophrenia, the dimensions with the highest scores were *help* and *avoidance*, with a mean score of 3.82 (SD = 0.82) and 3.31 (SD = 0.74) points, respectively, while *anger* was the dimension with the lowest score, with 1.75 (SD = 0.70) points. Police officers showed more willingness to help and greater feelings of pity and were less likely to consider psychiatric patients to be responsible for their actions when compared to Pedro whose mental health status was unknown (*p* <  0.001). They also showed more reactions of avoidance, considered mentally ill Pedro to be more dangerous, and saw a greater need to isolate him and coerce him into receiving medical treatment (*p* <  0.001). Post hoc paired Bonferroni comparisons (no mental illness vs schizophrenia or depression) revealed significant differences for all the AQ factors except for *anger* and *credibility* dimensions. The comparison between schizophrenia and depression revealed significant differences for the *dangerousness, coercion,* and *segregation* dimensions (*p* = 0.038, *p* = 0.002 and *p* = 0.011, respectively).

**Table 3:** Attribution Questionnaire factor scores according to psychiatric diagnosis

Factors/N° items	Schizophrenia Mean (SD) (***n*** = 312)	Depression Mean (SD) (***n*** = 302)	No mental illness Mean (SD) (***n*** = 290)	P^*^
Responsibility	2.00 (0.57)^n^	2.08 (0.61)^n^	2.29 (0.64)^s,d^	< 0.001
Pity	3.09 (0.72)^n^	3.04 (0.76)^n^	2.70 (0.82)^s,d^	< 0.001
Anger	1.75 (0.70)	1.72 (0.73)	1.62 (0.73)	0.08
Dangerousness	2.56 (0.58)^d,n^	2.45 (0.58)^s,n^	2.24 (0.62)^s,d^	< 0.001
Help	3.82 (0.82)^n^	3.81 (0.88)^n^	3.58 (0.95)^s,d^	< 0.001
Coercion	3.20 (0.85)^d,n^	3.01 (0.92)^s,n^	2.22 (0.87)^s,d^	< 0.001
Segregation	2.55 (0.74)^d,n^	2.37 (0.79)^s,n^	1.96 (0.77)^s,d^	< 0.001
Avoidance	3.31 (0.74)^n^	3.24 (0.76)^n^	3.01 (0.80)^s,d^	< 0.001
Credibility	2.95(0.63)	3.02(0.64)	3.06 (0.64)	0.102

*SD* standard deviation, *s* schizophrenia, *d* depression, *n* no mental illness

* One-way ANOVA; *p* <  0.05

* Superscripts^n,d,s^ indicate Bonferroni post hoc significant paired comparisons

### Association between vignette role and AQ scores

Table 4 shows the modified AQ dimension scores according to each hypothetical situation. *Help* was the construct with the highest scores while *anger* had the lowest score. Comparative one-way ANOVA tests of each factor in the different vignette roles revealed statistically significant differences in all AQ dimensions except for the *responsibility* construct. In order to see where the differences occurred, post hoc Bonferroni comparisons (assistance vs. victim vs. witness vs. suspect), were conducted.

**Table 4:** Attribution Questionnaire factors scores according to the vignette role

Factors^,^	Assistance Mean (SD) (*n* = 262)	Victim Mean (SD) (*n* = 239)	Witness Mean (SD) (*n* = 215)	Suspect Mean (SD) (*n* = 211)	P^*^
Responsibility	2.08 (0.64)	2.10 (0.58)	2.12 (0.62)	2.19 (0.62)	0.305
Pity	3.31 (0.7) ^w,s^	2.77 (0.74)	2.74 (0.76)^a^	2.86 (0.79)^a^	< 0.001
Anger	1.54 (0.63)^v,s^	1.77 (0.76)^a^	1.67(0.66)	1.83 (0.79) ^a^	< 0.001
Dangerousness	2.47(0.56) ^w^	2.35 (0.63) ^s^	2.26 (0.58) ^a,s^	2.56 (0.64) ^a,w^	< 0.001
Help	3.99 (0.84) ^v,w,s^	3.58 (0.86)^a^	3.64 (0.93)^a^	3.68 (0.86)^a^	< 0.001
Coercion	3.09 (0.92)^v,w^	2.76 (0.89)^a^	2.44 (0.97)^a,s^	2.91 (1.00) ^w^	< 0.001
Segregation	2.34 (0.70)^w,s^	2.31 (0.81)^w,s^	1.95 (0.75)^a,v,s^	2.55 (0.85)^a,v^	< 0.001
Avoidance	3.20 (0.74)^w,s^	3.15 (0.79)^w,s^	2.95 (0.75)^a,v,s^	3.44 (0.74)^a,v,w^	< 0.001
Credibility	3.16 (0.68)^v,s^	2.84 (0.59)^a,w^	3.26 (0.58)^s,v^	2.72 (0.49)^a,w^	< 0.001

*SD* standard deviation, *a* assistance, *v* victim, *w* witness, *s* suspect

*One-way ANOVA

* *p* < 0.05. Superscripts^a,v,w,s^ indicate Bonferroni post hoc significant paired comparisons

## Discussion

We believe this study is important because it is the first to specifically evaluate the phenomenon of evident stigma associated with mental illness shown among the Spanish State Security Forces. Since Watson’s research into the phenomenon of stigma in the American police, using the modified AQ questionnaire [17, 25], no previous study has applied this tool to the Spanish police, which gives our research added value.

In step two we analyzed stigmatizing attitudes and beliefs about mental illness with AQ questionnaire according to officers’ sociodemographic and working characteristics. Our findings indicate that female officers showed more feelings of pity and an increased desire to help mentally ill people than male officers. These results seem to support the conclusions of surveys carried out among the general population that suggest that women tend to have less stigmatizing attitudes towards mental illness than men [29]. Younger officers express a greater perception of danger and an increased desire to avoid psychiatric patients than older officers. These findings are consistent with previous research that suggests that experienced officers hold relatively less stigmatizing attitudes [22]. In contrast, research carried out among the general population concludes that older people tend to have more stigmatizing attitudes [30]. These differences may be due to the fact that younger officers, in their early years in the police force, tend to be more biased towards psychiatric patients suffering acute symptoms. These encounters in crisis situations may produce a misperception of danger, an attitude which may change for the better over the years after having experienced interactions with mentally ill individuals in a wider range of situations. We also found that the length of service was positively correlated with the *responsibility* dimension and negatively correlated with *avoidance* dimension. As previous studies have shown that training programs may reduce stigma [14, 31–33], we believe that the fact that experienced officers tend to consider psychiatric patients to be more responsible for their situation results from a lack of accurate knowledge about mental illness, as we included a wide range of variables for length of service and number of training programs in our sample.

In steps three and four, we analyzed the association between psychiatric history and stigmatizing attitudes. Our hypothesis was that mental health stigma would be evident regardless of the role of the psychiatric patient during interaction with police officers. MANOVA tests indicated significant main effects for psychiatric history and type of mental illness. No significant role-by-label interaction effects were found, so our hypothesis that stigma would be present regardless of the role, was supported. Subsequent ANOVA tests revealed significant main effects for psychiatric label on all AQ factors but the *anger* and *credibility* factors. We found increased desire for avoidance, greater perception of danger, and a stronger desire to segregate and coerce the psychiatric patients into receiving medical treatment, so our hypothesis about *dangerousness, avoidance, coercion* and *segregation* was supported. In addition, we evaluated the possible influence of two different disorders (schizophrenia and depression) on police encounters. We predicted that stigma would be worse for schizophrenia than for depressive disorder and would manifest as a stronger likelihood to isolate the person and to insist on treatment and, also, to avoid the person. According to our results, a diagnosis of schizophrenia increased the perception of danger and the desire to segregate and to coerce the subject into receiving medical treatment more than a diagnosis of depression. The differences between responses to schizophrenia and depressive disorder may be explained by the fact that the stereotype of dangerousness is more deeply ingrained in schizophrenia than in depression [34, 35]. With respect to the *avoidance* factor, which is an indicator of social distance, we found no significant differences between the two diagnoses, so our hypothesis was partially supported. Although the officers showed more positive attitudes towards depressive disorders, avoidance behaviors are one of the elements most related to stigmatization. Therefore, according to our findings, we cannot conclude that a diagnosis of depression always generates less stigma among police officers than schizophrenia, as studies carried out among the general population have suggested [6, 35–37].

Scores on *dangerousness*, *coercion,* and *segregation* factors were higher for psychiatric patients (especially those with schizophrenia), than for individuals whose mental status is unknown. The result for *dangerousness* suggests that this stereotype is intrinsically linked to a diagnosis of mental illness. Although the information provided about the type of mental illness and the vignette role may be different, the perception of *dangerousness* in the police context does not vary. These findings are consistent with those found in various police institutions around the world, where researchers argue that the most common police misconception is that all mentally ill people are dangerous [12, 17, 18, 20, 22, 38]. Unfortunately, the desire for segregating and coercing a subject into receiving medical treatment, together with the perception of dangerousness, may lead police officers to inadvertently escalate situations by approaching patients with threatening body language and speech, thereby provoking unnecessary violence in police encounters [17].

Police officers were not likely to experience anger in mental health-related encounters and they did not consider psychiatric patients to be less credible than someone whose mental health status was unknown. Perhaps the most positive finding to highlight in the comparative analysis between people with and without mental illness is that our hypothesis about *credibility* was not supported: psychiatric history was not associated with lower perceived *credibility*. Individuals with a psychiatric diagnosis may be viewed as untrustworthy and unable to provide reliable information, so they are particularly vulnerable to victimization [17]. If they seek assistance from police officers, our findings indicated that they may be taken seriously and provided with the assistance they need.

We predicted that the label of mental illness would increase a police officer’s willingness to help the person, would evoke feelings of pity, and would make the person seem less responsible for his or her actions. Our findings partially supported these hypotheses because in the comparative analysis between schizophrenia and depression, the *responsibility* dimension did not reveal any significant differences. This finding is likely related to whether an officer is already aware that depressive disorders are in fact, mental illnesses. One of the greatest problems suffered by people with depression is that they may be considered weak and responsible for their situation because of the stereotypes among the general population that perpetuate this notion [34, 39–42].

In general, our results are consistent with those of Watson [17]. When comparing the scores for each factor according to whether the person had a mental illness or not, the differences were low (< 0.5 points), except for the *pity* and *coercion* dimensions, where Watson et al. obtained lower scores than we did. These results could be due to the fact that we included two types of mental illnesses in our study (schizophrenia and depression) each of which evokes different degrees of stigma [35]. The differences in the *pity* dimension may highlight police attitudinal changes over time or cultural differences, as some studies suggest that there exists less stigma among the Spanish population [43].

In step five, we analyzed the association between vignette role and stigmatizing attitudes. Interaction effects of mental illness and vignette role were not significant in MANOVA tests. ANOVA results indicated significant main effects for role on all nine subscales of the AQ except for *responsibility*. *Help* was the construct with the highest scores in all roles while *anger* had the lowest score regardless of the mental status. When comparing our results with Watson et al., they found significant differences in *responsibility*. Our results seem to support the conclusions of attribution theory that suggests that emotion (anger or sympathy) mediates cognition (attribution and judgment of responsibility) and action (helping or punishing behavior) [44, 45]. Police officers may perceive the cause of the situation as uncontrollable, and judge the person as not responsible, not experiencing anger, and trying to help the subject regardless the role he or she is playing.

Some limitations should be considered when interpreting our results. The vignettes did not include certain variables, such as Pedro’s behavior, that are usually present in daily law enforcement situations. Further studies should evaluate these results in other situations more representative of daily police practice. A second limitation is that we explored the phenomenon of stigma with a self-administered questionnaire. Although these questionnaires have proven to be practical and cost-effective, with low participant burden, they have limitations in terms of recall bias and social desirability bias [46]. In addition, the research was cross-sectional, so reverse causation and certain degree of residual confounding cannot be ruled out. Another limitation arises from the fact that personal factors that may influence police attitudes have not been controlled for, as the objective of this study was to assess the influence of the stigma of mental illness among police officers. As the results are intended for training purposes, these unresolved issues should be better assessed in future studies.

The greatest strength of this research lies in the fact that it should help to provide valuable information for improving law enforcement protocols among the Spanish police forces during interactions with psychiatric patients and may also help to guide and update police training. Recent studies carried out among police forces in other countries have shown that specific training programs about mental health issues help to reduce stigmatizing attitudes and increase understanding and support for people diagnosed with a psychiatric disorder [14, 31–33].

## Conclusions

Our findings highlight several issues that should be addressed within the police force. Although police officers are generally aware of mental illness, they often hold negative attitudes and beliefs that require attention. Our study provides evidence that labeling a subject with a mental illness, especially when the diagnosis is schizophrenia, produces some unwanted effects on decision-making within law enforcement agencies. We are able to tease out specific factors that increased stigma and that will be helpful in the design of police training programs. We recommend that such training begins early in a police officer’s career. Patients with a severe psychiatric illness may be more vulnerable than others when interacting with the police in the sense that their speech and behaviour may be misunderstood [47]. Improving law enforcement training and protocols should be able to reduce manifestations on the part of police of unwarranted bias and stigma.

## Data Availability

The datasets generated and analyzed during the current study are not publicly available on request from the corresponding author. The data are not publicly available due to privacy restrictions (include information that allows the identification of members of the state security forces) but are available from the corresponding author upon reasonable request.

## Abbreviations

AQ-27: Attribution Questionnaire-27
SD: standard deviation

## References

[1] Šiška J, Beadle-Brown J. Report on the transition from institutional care to community-based services in 27 Eu member states. 2020. https://deinstitutionalisationdotcom.files.wordpress.com/2017/11/report-fo-the-ad-hoc_2009.pdf. Accessed 9 Aug 2022.

[2] Angermeyer MC, Carta MG, Ghachem R, Matschinger H, Millier A, Refai T, et al. Cultural Variations in Public Beliefs about Mental Disorders: A Comparison between Tunisia and Germany. Clin Pract Epidemiol Ment Health. 2020;16(Suppl-1):70–81. 10.2174/1745017902016010070 PMC7536730 33029184

[3] Husain MO, Zehra SS, Umer M, Kiran T, Husain M, Soomro M (2020). Stigma toward mental and physical illness: attitudes of healthcare professionals, healthcare students and the general public in Pakistan. B J Psych Open.

[4] Oliveira AM, Machado D, Fonseca JB, Palha F, Silva Moreira P, Sousa N (2020). Stigmatizing attitudes toward patients with psychiatric disorders among medical students and professionals. Front Psychiatry..

[5] Martinelli TF, Meerkerk GJ, Nagelhout GE, Brouwers EPM, van Weeghel J, Rabbers G (2020). Language and stigmatization of individuals with mental health problems or substance addiction in the Netherlands: an experimental vignette study. Heal Soc Care Community.

[6] Mannarini S, Rossi A, Munari C. How do education and experience with mental illness interact with causal beliefs, eligible treatments and stigmatising attitudes towards schizophrenia? A comparison between mental health professionals, psychology students, relatives and patients. BMC Psychiatry. 2020;20:167. 10.1186/s12888-020-02580-6 PMC7161111 32295581

[7] Paananen J, Lindholm C, Stevanovic M, Weiste E (2020). Tensions and paradoxes of stigma: discussing stigma in mental health rehabilitation. Int J Environ Res Public Health.

[8] da Silva AG, Baldaçara L, Cavalcante DA, Fasanella NA, Palha AP (2020). The impact of mental illness stigma on psychiatric emergencies. Front Psychiatry.

[9] Kesic D. The role of mental disorders in use of force incidents between the police and the public. In: Policing and the mentally ill: international perspectives: Taylor and Francis; 2013. p. 153–70.

[10] Ogloff JR, Thomas SD, Luebbers S, Baksheev G, Elliott I, Godfredson J (2013). Policing services with mentally ill people: developing greater understanding and best practice. Aust Psychol.

[11] Puntis S, Perfect D, Kirubarajan A, Bolton S, Davies F, Hayes A (2018). A systematic review of co-responder models of police mental health “street” triage. BMC Psychiatry..

[12] Soares R, Da Costa MP. Experiences and perceptions of police officers concerning their interactions with people with serious mental disorders for compulsory treatment. Front Psychiatry. 2019;10 MAR:187. 10.3389/fpsyt.2019.00187 PMC6482210 31057433

[13] Godfredson JW, Thomas SDM, Ogloff JRP, Luebbers S (2011). Police perceptions of their encounters with individuals experiencing mental illness: a Victorian survey. Aust New Zeal J Criminol.

[14] Wood JD, Watson AC (2017). Improving police interventions during mental health-related encounters: past, present and future. Polic Soc.

[15] Queensland Interagency Agreement for the Safe Transport of People Accessing Mental Health Assessment , Treatment and Care 2019 Queensland Health ( including the Queensland Ambulance Service and Retrieval Services Queensland ) Queensland Police Service for. 2019.

[16] Protocolo de coordinación de actuaciones para los traslados e ingresos de personas que padecen enfermedad mental. Servicio Murciano de Salud 2007;:19. https://sms.carm.es/ricsmur/handle/123456789/4526?show=full. Accessed 9 Aug 2022.

[17] Watson AC, Corrigan PW, Ottati V (2004). Police officers’ attitudes toward and decisions about persons with mental illness. Psychiatr Serv.

[18] Chen C, Ou JJ, Zhou JS, Zhang YD, Cai WX, Wang XP (2013). The comparison of disposal attitudes towards forensic psychiatric patients among police officers, psychiatrists and community members in China. J Forensic Legal Med.

[19] Cotton D (2004). The attitudes of Canadian police officers toward the mentally ill. Int J Law Psychiatry.

[20] Ruiz J, Miller C (2004). An exploratory study of Pennsylvania police officers’ perceptions of dangerousness and their ability to manage persons with mental illness. Police Q.

[21] Litzcke SM (2006). Attitudes and emotions of German police officers towards the mentally ill. Int J Police Sci Manag.

[22] Psarra V, Sestrini M, Santa Z, Petsas D, Gerontas A, Garnetas C (2008). Greek police officers’ attitudes towards the mentally ill. Int J Law Psychiatry.

[23] Siu BWM, Chow KKW, Lam LCW, Chan WC, Tang VWK, Chui WWH (2012). A questionnaire survey on attitudes and understanding towards mental disorders. East Asian Arch Psychiatr.

[24] Corrigan P, Markowitz FE, Watson A, Rowan D, Kubiak MA (2003). An attribution model of public discrimination towards persons with mental illness. J Health Soc Behav.

[25] Watson AC, Corrigan PW, Ottati V (2004). Police responses to persons with mental illness: does the label matter?. J Am Acad Psychiatry Law..

[26] Pingani L, Forghieri M, Ferrari S, Ben-Zeev D, Artoni P, Mazzi F (2012). Stigma and discrimination toward mental illness: translation and validation of the Italian version of the attribution questionnaire-27 (AQ-27-I). Soc Psychiatry Psychiatr Epidemiol.

[27] Akyurek G, Efe A, Kayihan H (2019). Stigma and discrimination towards mental illness: translation and validation of the Turkish version of the attribution Questionnaire-27 (AQ-27-T). Community Ment Health J.

[28] Muñoz M, Guillén AI, Pérez-Santos E, Corrigan PW (2015). A structural equation modeling study of the Spanish mental illness stigma attribution questionnaire (AQ-27-E). Am J Orthop.

[29] Angermeyer MC, Buyantugs L, Kenzine DV, Matschinger H (2004). Effects of labelling on public attitudes towards people with schizophrenia: are there cultural differences?. Acta Psychiatr Scand.

[30] TNS-BMRB. Attitudes to mental illness 2014 research report. Prepared for time to change. 2015. https://www.bl.uk/collection-items/attitudes-to-mental-illness-2014-research-report. Accessed 9 Aug 2022.

[31] Warner R (2005). Local projects of the world psychiatric association Programme to reduce stigma and discrimination. Psychiatr Serv.

[32] Rogers MS, McNiel DE, Binder RL (2019). Effectiveness of police crisis intervention training programs. J Am Acad Psychiatry Law.

[33] Booty D, M, G Williams R, K Crifasi C. (2020). Evaluation of a crisis intervention team pilot program: results from Baltimore, MD. Community Ment Health J.

[34] Kelly CM, Jorm AF (2007). Stigma and mood disorders. Curr Opinion Psychiatry.

[35] Angermeyer MC, Matschinger H (2003). The stigma of mental illness: effects of labelling on public attitudes towards people with mental disorder. Acta Psychiatr Scand.

[36] Nordt C, Rössler W, Lauber C (2006). Attitudes of mental health professionals toward people with schizophrenia and major depression. Schizophr Bull.

[37] Griffiths KM, Nakane Y, Christensen H, Yoshioka K, Jorm AF, Nakane H (2006). Stigma in response to mental disorders: a comparison of Australia and Japan. BMC Psychiatry..

[38] Bell S, Palmer-conn S. Suspicious minds : police attitudes to mental ill health suspicious minds : police attitudes to mental ill health. Int J Law Public Adm. 2018;1 February 2019:25–40.

[39] Coppens E, Van Audenhove C, Scheerder G, Arensman E, Coffey C, Costa S (2013). Public attitudes toward depression and help-seeking in four European countries baseline survey prior to the OSPI-Europe intervention. J Affect Disord.

[40] Schomerus G, Schwahn C, Holzinger A, Corrigan PW, Grabe HJ, Carta MG (2012). Evolution of public attitudes about mental illness: a systematic review and meta-analysis. Acta Psychiatr Scand.

[41] Angermeyer MC, Matschinger H (2004). Public attitudes to people with depression: have there been any changes over the last decade?. J Affect Disord.

[42] Angermeyer MC, Matschinger H (2003). Public beliefs about schizophrenia and depression: similarities and differences. Soc Psychiatry Psychiatr Epidemiol.

[43] Bengochea-Seco R, Arrieta-Rodríguez M, Fernández-Modamio M, Santacoloma-Cabero I, Gómez de Tojeiro-Roce J, García-Polavieja B (2018). Adaptation into Spanish of the internalised stigma of mental illness scale to assess personal stigma. Rev Psiquiatr. Salud Ment.

[44] Corrigan PW (2000). Mental health stigma as social attribution: implications for research methods and attitude change. Clin Psychol Sci Pract.

[45] Schmidt G, Weiner B (1988). An attribution-affect-action theory of behavior: replications of judgments of help-giving. Personal Soc Psychol Bull.

[46] Hamann J, Kissling W, Mendel R (2016). Does it matter whether physicians’ recommendations are given early or late in the decision-making process? An experimental study among patients with schizophrenia. BMJ Open.

[47] Charette Y, Crocker AG, Billette I (2011). The judicious judicial dispositions juggle: characteristics of police interventions involving people with a mental illness. Can J Psychiatr.

